# Alcohol Use Disorder and Depression: The Complexity of Comorbidity

**DOI:** 10.1192/j.eurpsy.2024.1097

**Published:** 2024-08-27

**Authors:** A. H. I. Abu Shehab, T. Simona, A. B. Ciubară, D. C. Voinescu, L. Burlea, A. Ciubară

**Affiliations:** ^1^Psychiatry Department, “Elisabeta Doamna” Psychiatric Hospital, Galati; ^2^Psychiatry Department, Carol Davila University of Medicine and Pharmacy, Bucharest; ^3^Orthopedics and traumatology Department; ^4^Rheumatology department, “Dunărea de Jos” University - Faculty of Medicine and Pharmacy, Galati; ^5^Technology, Prothesis and Implantology, University of Medicine and Pharmacy “Grigore T. Popa”, Iași; ^6^Psychiatry Department, “Dunărea de Jos” University - Faculty of Medicine and Pharmacy, Galati, Romania

## Abstract

**Introduction:**

Alcohol Use Disorder (AUD) and depression are among the most prevalent mental health concerns on a global scale. The co-occurrence of alcohol use disorder (AUD) and depression has been well acknowledged, leading to intricate issues in diagnosis, treatment, and prognosis.

**Objectives:**

This study aims to analyse the complex correlation between AUD (Alcohol Use Disorder) and depression, with a specific emphasis on examining common underlying causes, reciprocal influences, and potential implications for clinical treatment.

**Methods:**

An exhaustive review of literature was undertaken, emphasizing epidemiological studies, neurobiological research, and the efficacy of combined treatment modalities. The review also delved into the potential role of genetics, environmental factors, and psychosocial stressors in co-occurrence.

**Results:**

The available evidence indicates that there exists a reciprocal relationship between depression and alcohol use disorder (AUD), wherein each disease can serve as a triggering factor for the other. This interplay between depression and AUD forms a detrimental cycle that intensifies the severity of both conditions. The comorbidity of various disorders may be attributed to the presence of shared neurochemical pathways, with a particular emphasis on the serotonin system. Furthermore, the co-occurrence of both illnesses frequently leads to heightened symptom severity, reduced treatment efficacy, and a higher risk of suicide.

**Conclusions:**

The complex relationship between alcohol use disorder (AUD) and depression underscores the need for a comprehensive and integrated therapy strategy. The effective management of this comorbidity necessitates the implementation of multidisciplinary collaboration, patient education, and early intervention.

**Disclosure of Interest:**

None Declared

